# Comparative osteohistology of *Hesperornis* with reference to pygoscelid penguins: the effects of climate and behaviour on avian bone microstructure

**DOI:** 10.1098/rsos.140245

**Published:** 2014-11-19

**Authors:** Laura E. Wilson, Karen Chin

**Affiliations:** Department of Geological Sciences, University of Colorado, Boulder, CO, USA

**Keywords:** *Hesperornis*, histology, penguins, high-latitude, Cretaceous, avian growth

## Abstract

The broad biogeographic distribution of *Hesperornis* fossils in Late Cretaceous Western Interior Seaway deposits has prompted questions about whether they endured polar winters or migrated between mid- and high latitudes. Here, we compare microstructures of hesperornithiform long bones from Kansas and the Arctic to investigate whether migration or Late Cretaceous polar climate affected bone growth. We also examine modern penguin bones to determine how migration and climate may influence bone growth in birds with known behaviours. Histological analysis of hesperornithiform samples reveals continuous bone deposition throughout the cortex, plus an outer circumferential layer in adults. No cyclic growth marks, zonation or differences in vasculature are apparent in the *Hesperornis* specimens. Comparatively, migratory Adélie and chinstrap penguin bones show no zonation or changes in microstructure, suggesting that migration is not necessarily recorded in avian bone microstructure. Non-migratory gentoos show evidence of rapid bone growth possibly associated with increased chick growth rates in high-latitude populations and large body size. The absence of histological evidence for migration in extinct *Hesperornis* and extant pygoscelid penguins may reflect that these birds reached skeletal maturity before migration or overwintering. This underscores the challenges of using bone microstructure to infer the effects of behaviour and climate on avian growth.

## 2. Introduction

**Figure 1.**
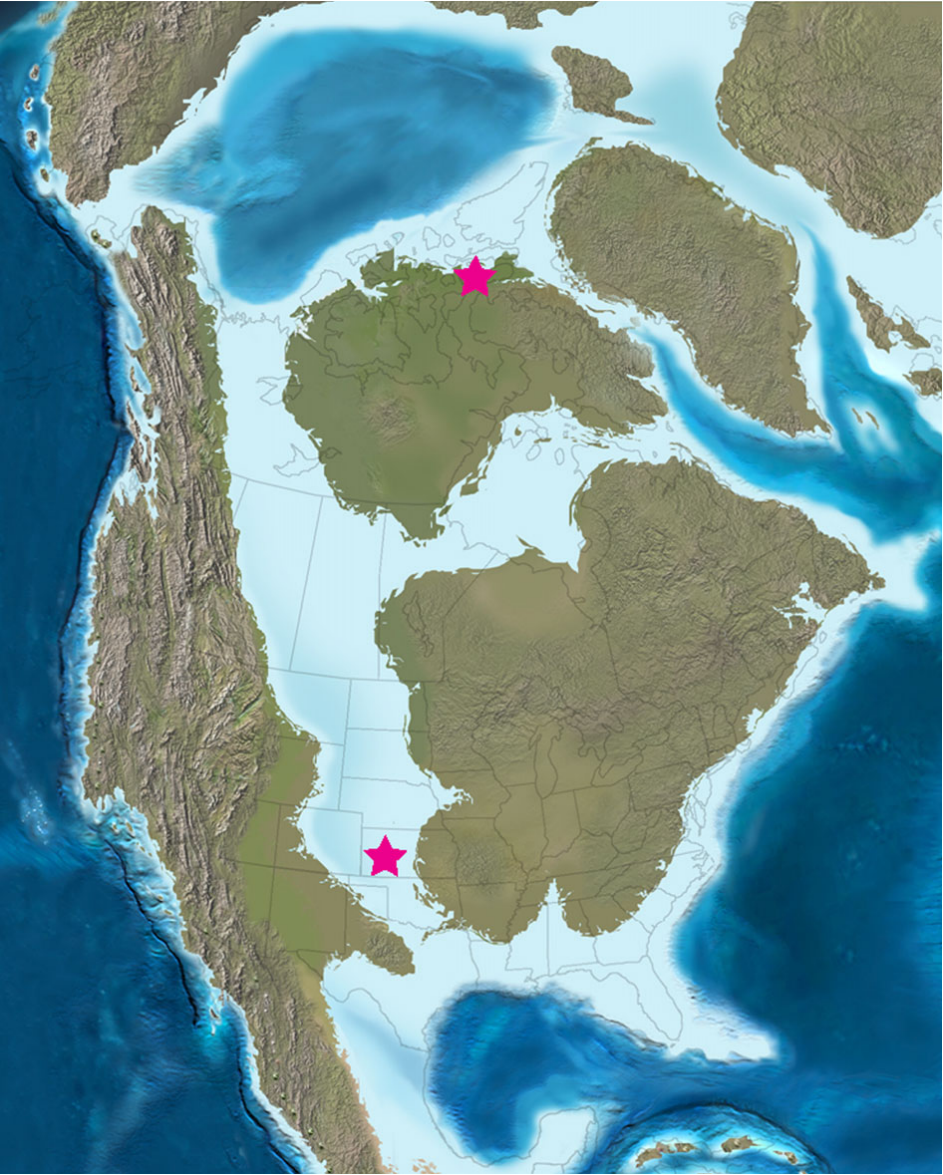
Map showing Late Cretaceous North American palaeogeography. Stars indicate specimen provenance of *Hesperornis* bones from the Niobrara Chalk of Kansas and the Kanguk Formation of Devon Island, Canadian High Arctic. Map modified from Blakey [13]. See table 1 for additional specimen information.

Hesperornithiforms were a group of flightless seabirds that inhabited Late Cretaceous marine environments throughout the Northern Hemisphere. They have a particularly good fossil record from the Campanian Western Interior Seaway (WIS) of North America, ranging from Arkansas, USA, to Ellesmere Island in the Canadian Arctic. This extensive biogeographic range indicates that hesperornithiforms were successful in a variety of environments [1–4]. The 33° N (Arkansas) [5] to 79° N (Ellesmere Island) [6] palaeolatitudinal distribution of *Hesperornis* fossils, coupled with an abundance of juvenile *Hesperornis* remains in the Arctic (and the absence of juveniles from lower latitudes) has led to the hypothesis that *Hesperornis* migrated along the WIS [2,6–8], perhaps to breed in higher latitude environments. These birds were highly adapted for foot-propelled diving [9–11], and given the secondary loss of flight, could have migrated by swimming north and south through the Seaway. Though this may seem remarkable, many modern penguin species are known to migrate thousands of kilometres during the non-breeding season [12]. Consequently, the discovery of *Hesperornis*at high latitudes not only raises questions about their palaeobiology in these polar environments, but provides an opportunity to explore the migration hypothesis by comparing specimens found in mid- and high-latitude fossil assemblages. Here, we describe and compare the bone microstructure of *Hesperornis* femora and tibiotarsi from Kansas to a cf. *Hesperornis*femur from Devon Island (Canadian High Arctic; figure 1) to look for evidence relating to long-distance migration or overwintering in Late Cretaceous high-latitude environments. To help interpret results, patterns of *Hesperornis* bone histology are compared with those of extant high-latitude penguins.

Late Cretaceous environments were characterized by greenhouse conditions [14–18], but strong seasonality related to temperature and photoperiodic variation probably influenced ancient polar ecosystems during this period [18–22]. Sea surface temperatures along the Late Cretaceous WIS are estimated to have been 22–24°C in the Gulf of Mexico [23] and between 10°C [14] and 15°C in the Arctic Ocean ([17], though this probably represents a summer sea surface temperature, see [21]). Estimates of Cretaceous Arctic ambient mean annual temperature range from between 8° and −2°C [24,25] to −5°C [17]. Despite evidence for seasonality and below freezing temperatures [17–19,22,24,26], it is unclear whether Late Cretaceous polar climates were variable enough to influence vertebrate growth dynamics. However, previous research suggests that strong seasonality in the Campanian Arctic may have affected body size evolution [3] of high-latitude hesperornithiforms.

Bone histology offers an effective tool for studying growth in extinct and extant vertebrates, as bone tissues reflect bone growth rates, thus preserving a record of change in growth dynamics through ontogeny [27–33]. Variations in growth patterns can be recorded by cyclic growth marks, such as zones, annuli and lines of arrested growth (LAGs) [28,34]. Cyclic growth marks like LAGs run parallel to the periosteal margin (circumferential), completely circling the bone; they do not erode primary or secondary osteons [35]. Annuli are regular deposits of bone tissue throughout the cortex, often characterized by reduced osteocyte and/or vascular canal density. Using this information, bone microstructure has recently been used to test hypotheses of migratory behaviour in non-avian dinosaurs [36–41]. Specifically, several studies have inferred a correlation between the presence or absence of growth marks in bones of high-latitude dinosaurs and the physiological stresses of long-distance migrations or overwintering in Arctic climates [36–41]. Such analyses have resulted in varying conclusions regarding the relationship between migration and overwintering and bone microstructure. In addition, these investigations focus on non-avian dinosaurs with little reference to modern analogues. Without a comprehensive understanding of how modern organisms grow with respect to ontogeny, biomechanical demands, behaviour, and environmental cues and stresses, interpretations of fossil bone microstructure are merely speculative.

Given the importance of comparative histological studies employing modern avian analogues, this study of fossil *Hesperornis* bones also considers the bone microstructure of *Pygoscelis* penguins (Adélies, chinstraps and gentoos). Though penguins are not functional analogues of hesperornithiforms in terms of locomotion (penguins are wing-propelled rather than foot-propelled divers), they are considered good ecological analogues given their biogeography and range of body sizes [42]. Known differences in pygoscelid penguin migratory behaviour and biogeographic distribution permit testing for bone microstructure patterns related to behaviour and environment and allow us to better interpret the presence or absence of patterns in *Hesperornis* bone histology. Studying modern analogues enables comparisons among populations for which behaviour and environmental conditions are known, thus leading to a better understanding of how various intrinsic and extrinsic factors affect bone growth.

## 3. Material and methods

Femora and tibiotarsi identified to the genus *Hesperornis* were analysed in this study; fossil material was included based on availability for destructive analysis, preservation of the mid-diaphysis of long bones and confidence in taxonomic identification. Specimens from the Niobrara Chalk of Kansas, USA (YPM 1201; YPM 1489; YPM 1491) and the Kanguk Formation of Devon Island, Nunavut, in the Canadian Arctic (NUVF 286) were thin sectioned for histological analysis (table 1). Eight other Arctic specimens identified as hesperornithiforms in museum collections were also sectioned (CMN 11409, CMN 11421, CMN 11441, CMN 40730, CMN 40824, CMN 41054, CMN 51580, CMN 51585), but taphonomic and taxonomic issues prevented their inclusion in this study. Microbial alteration obscured the microstructure of three of these bones, and the other five specimens could not be confidently attributed to *Hesperornis* or even Aviale. Thus poor preservation and the rare and fragmentary nature of Arctic fossils restricted the Arctic sample to one femur. NUVF 286 is the right femur of a bird identified as cf. *Hesperornis* sp. [4]. The three Kansas specimens have also been identified as *Hesperornis* and include one femur (YPM 1201) and two tibiotarsi (YPM 1489, YPM 1491) from separate individuals.

**Table 1.** Specimen and collection information for bones used in histological analysis.

specimen number	specimen ID	element	locality	ontogenetic stage
YPM 1201	*Hesperornis regalis*	femur	Niobrara Chalk, KS, USA	adult
YPM 1489	*Hesperornis* sp.	tibiotarsus	Niobrara Chalk, KS, USA	adult
YPM 1491	*Hesperornis regalis*	tibiotarsus	Niobrara Chalk, KS, USA	adult
NUVF 286	*Hesperornis* sp.	femur	Kanguk Fm., Devon Island, Canada	sub-adult
UCM 104148	Adélie penguin (*Pygoscelis adeliae*)	femur, tibiotarsus	Seymour Island, Antarctica (64°S)	adult
UNCW B963	chinstrap penguin (*Pygoscelis antarctica*)	femur, tibiotarsus	Meade Island, Antarctica (62°S)	adult
UNCW B980	gentoo penguin (*Pygoscelis papua*)	femur, tibiotarsus	Aitcho Island, Antarctica (62°S)	adult

Modern *Pygoscelis* penguin (Adélie, chinstrap and gentoo) bones were also thin sectioned. Femora and tibiotarsi from three Adélie (*Pygoscelis adeliae*), four chinstrap (*Pygoscelis antarctica*) and three gentoo (*Pygoscelis papua*) individuals (naturally deceased) from the Antarctic region were examined (table 1; electronic supplementary material). Because the birds were already deceased, partially disarticulated and mostly defleshed at the time of collection, the sex of the individuals is not known. *Pygoscelis* penguin bones were collected from breeding colonies on Antarctica, the Antarctic Peninsula and the islands surrounding the Antarctic Peninsula (tables 1 and 2) by Dr Steve Emslie and his students (University of North Carolina Wilmington), Dr David Ainley (H. T. Harvey & Associates) and Dr Kate Dugger (Oregon State University) for the purpose of histological analysis.

**Table 2.** Pygoscelid penguin biology, ecology and behaviour.

species	biogeographic range (° S)	asymptotic size (g)	migratory behaviour	growth constant^*a*^	vascular canal density (canals mm^−2^)
Adélie (*Pygoscelis adeliae*)	54–77 [12]	3940 [43]	migratory	0.146 [43]	femur: 35.7
					tibiotarsus: 31.4
chinstrap (*Pygoscelis antarctica*)	54–69 [12]	4025 [43]	migratory	0.127 [43]	femur: 35.2
					tibiotarsus: 32.5
gentoo (*Pygoscelis papua*)	46–65 [12,44]	5725 [43]	non-migratory	0.071–0.113 [43,45]	femur: 52.9
					tibiotarsus: 44.4

^*a*^Maximum slope of growth curve between 10 and 90% of adult size; *sensu*[46].

Thin sections of fossil material were prepared using the standard procedure of Wilson [47] and Lamm [48,49]. All specimens were photographed (see Morphobank project P1270), measured (see the electronic supplementary material), moulded and cast prior to sectioning. Bones were embedded in a two-part epoxy resin and hardener (Struers Epofix), and multiple slices of each embedded bone were cut through the diaphysis with a low-speed circular saw (Buehler Isomet) with a 5 inch diameter diamond-edged Norton saw blade. Cut sections were mounted on frosted glass slides with 2 ton epoxy (Devcon) and then ground on a lap wheel (Buehler Ecomet III grinder/polisher) with 400, 600 and 800 grit carbimet abrasive papers to desired thickness for histological analysis. Finally, thin sections were polished with 5 μm alumina powder for optical clarity.

Additional steps were taken for sectioning modern bones. Prior to sectioning, modern bones were soaked in a 10% buffered (sodium phosphate) formalin solution for up to a week (depending on the amount of soft tissue present). After a week in solution, a mid-diaphyseal section was removed from each bone using the Isomet circular saw. Cut bone sections were defatted in diluted stain remover (Zout) and dehydrated in graded ethanol solutions (70 and 85% for 2 days each, 100% for up to a week) prior to embedding in resin. Embedding, mounting, grinding and polishing followed the same methodology as described for fossil bones.

Two to four transverse sections were made through the diaphysis of all bones in this study and interpretations are based on observations in that plane. All thin sections were analysed and photographed using a Leica DMR microscope and a SPOT RT Slider digital camera system. Images were compiled using ImageJ software (with the MosaicJ plugin) and edited in Photoshop. Temporary glass coverslips were applied to some thin sections with immersion oil for microscopy. High-resolution images of complete tranverse sections, images figured in this study and additional images are reposited under project P1270 at Morphobank (http://morphobank.org).

### 3.1 Vascular canal densities

Vascular canal density was calculated for the modern penguin specimens included in this study. This variable was measured as the number of vascular canals divided by the primary bone area (mm^2^). Femoral and tibiotarsal canal densities from each individual specimen were averaged for each species.

### 3.2 Institutional abbreviations

CMN: Canadian Museum of Nature, Ottawa, Canada; NUVF: Nunavut vertebrate fossil collection (housed at CMN, Ottawa, Canada); UCM: University of Colorado Museum of Natural History (Paleontology Section), Boulder, CO, USA; UNCW: University of North Carolina, Wilmington Ornithology Collection, Wilmington, NC, USA; YPM: Yale Peabody Museum, New Haven, CT, USA.

## 4. Histological descriptions

Previous investigations have described the microstructure of *Hesperornis* bones from the Niobrara Chalk of Kansas, but to date, no studies have analysed the histology of hesperornithiform specimens from high-latitude deposits. Houde [50] considered the phylogenetic usefulness of bone microstructure for determining the systematic position of *Hesperornis*, primarily with regard to vascular canal patterns. Subsequent studies, however, have emphasized that ontogeny, function and environment also affect bone microstructure [27,51–57]. Chinsamy [58] found microstructure patterns in *Hesperornis* bones to be consistent with those of modern penguins, emphasizing the high metabolic capabilities and adaptations to flightlessness and diving (specifically, thickened cortical bone) in *Hesperornis*.

**Figure 2.**
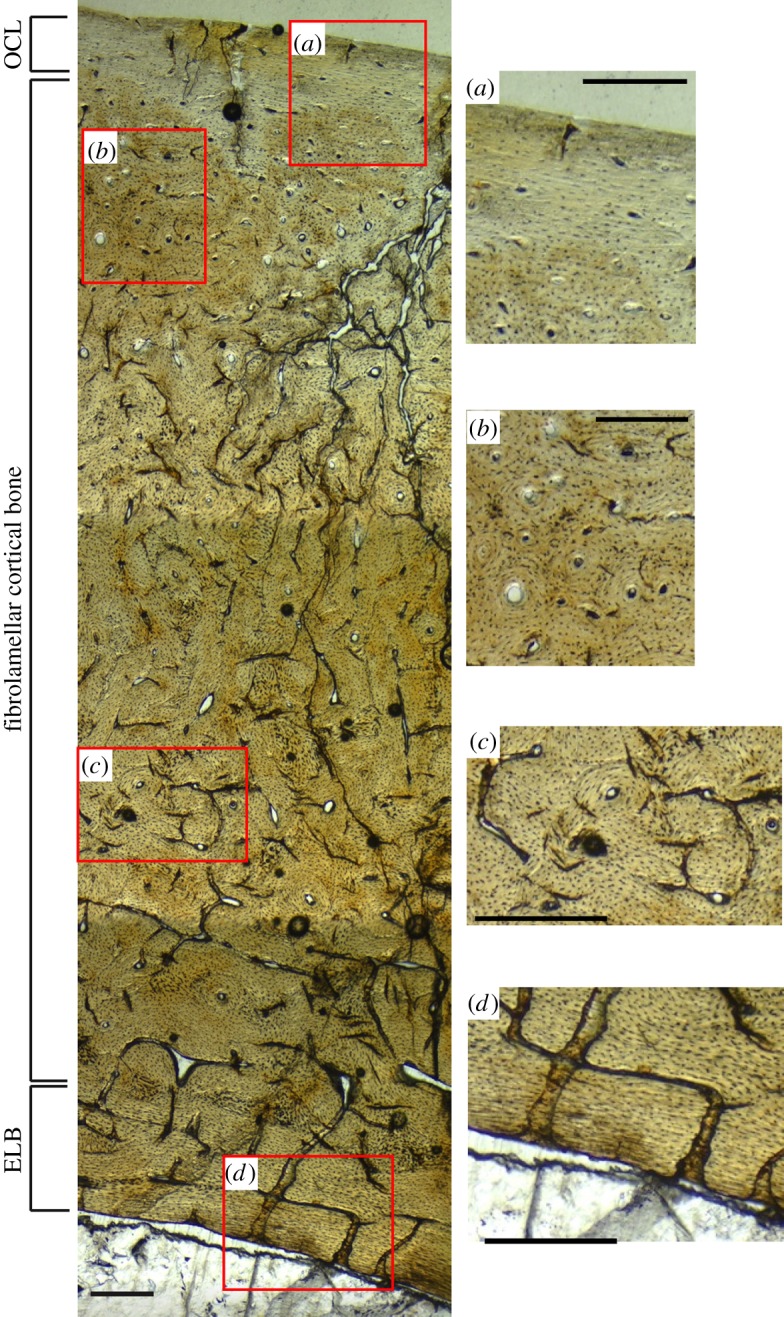
*Hesperornis* tibiotarsus (YPM 1491) illustrating the three layers characterizing adult avian bone microstructure. Section through transverse plane of the bone. Close-ups show (*a*) transition from the OCL to the fibrolamellar cortex, (*b*) longitudinal bone, (*c*) reticular bone and (*d*) transition from the fibrolamellar cortex to ELB. ELB, endosteal lamellar bone; OCL, outer circumferential layer. Scale bar, 250 μm.

**Figure 3.**
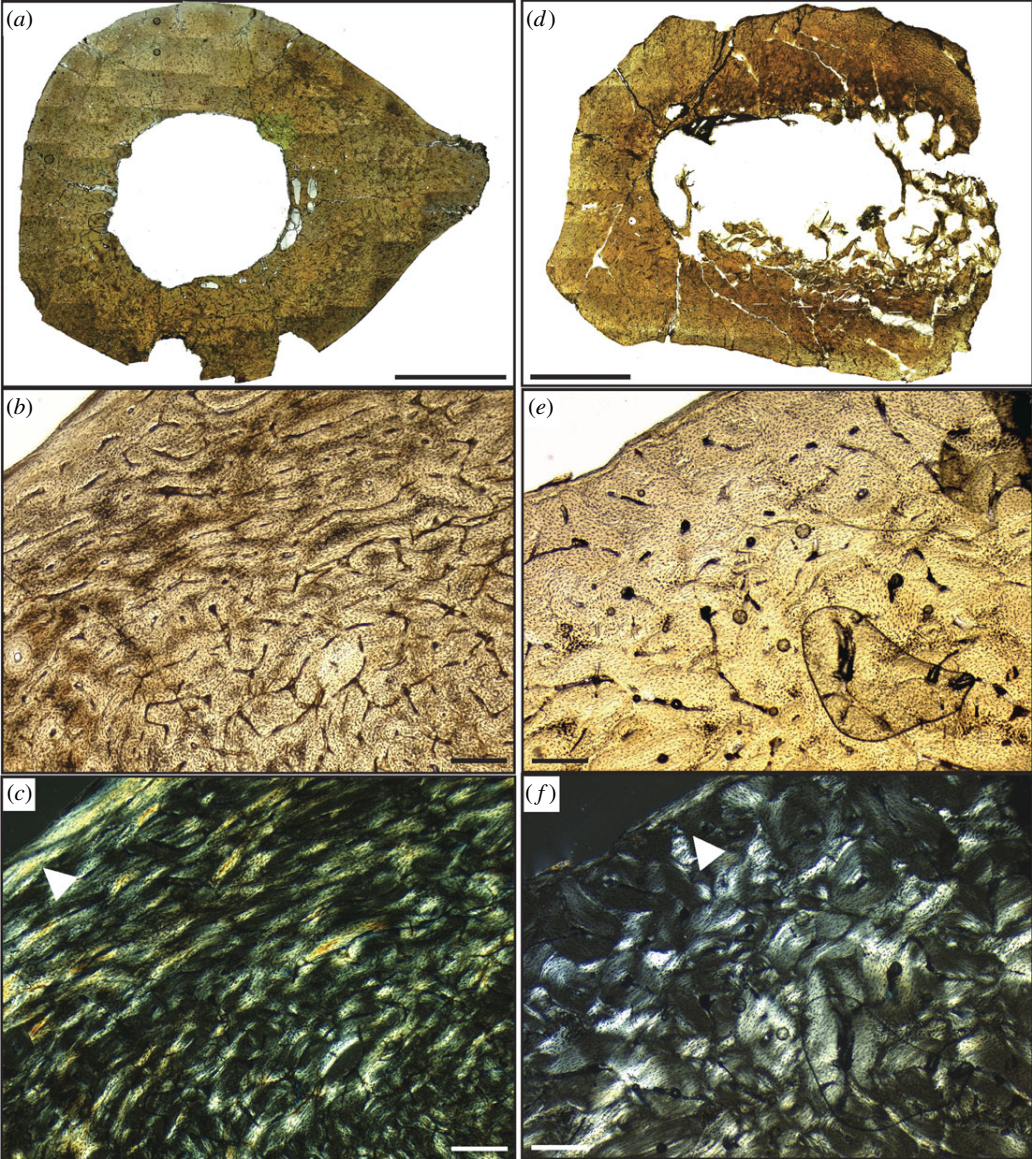
Bone microstructure of (*a*–*c*) Kansas *Hesperornis* tibiotarsus YPM 1491 and (*d*–*f*) Kansas *Hesperornis* femur YPM 1201. (*a*) Composite transverse section of tibiotarsus showing shape and general histology patterns. (*b*) Close-up of cortical bone microstructure in plain light. (*c*) Same view as (*b*) but in cross-polarized light to show collagen fibre orientation. White arrow indicates the OCL along the periosteal surface. (*d*) Composite transverse section of femur showing shape and general histology patterns. (*e*) Close-up of cortical bone microstructure in plain light. (*f*) Same view as (*e*) but in cross-polarized light to show collagen fibre orientation. White arrow indicates the OCL along the periosteal surface. Scale bar, (*a*,*d*) 5 mm and (*b*,*c*,*e*,*f*) 250 μm.

**Figure 4.**
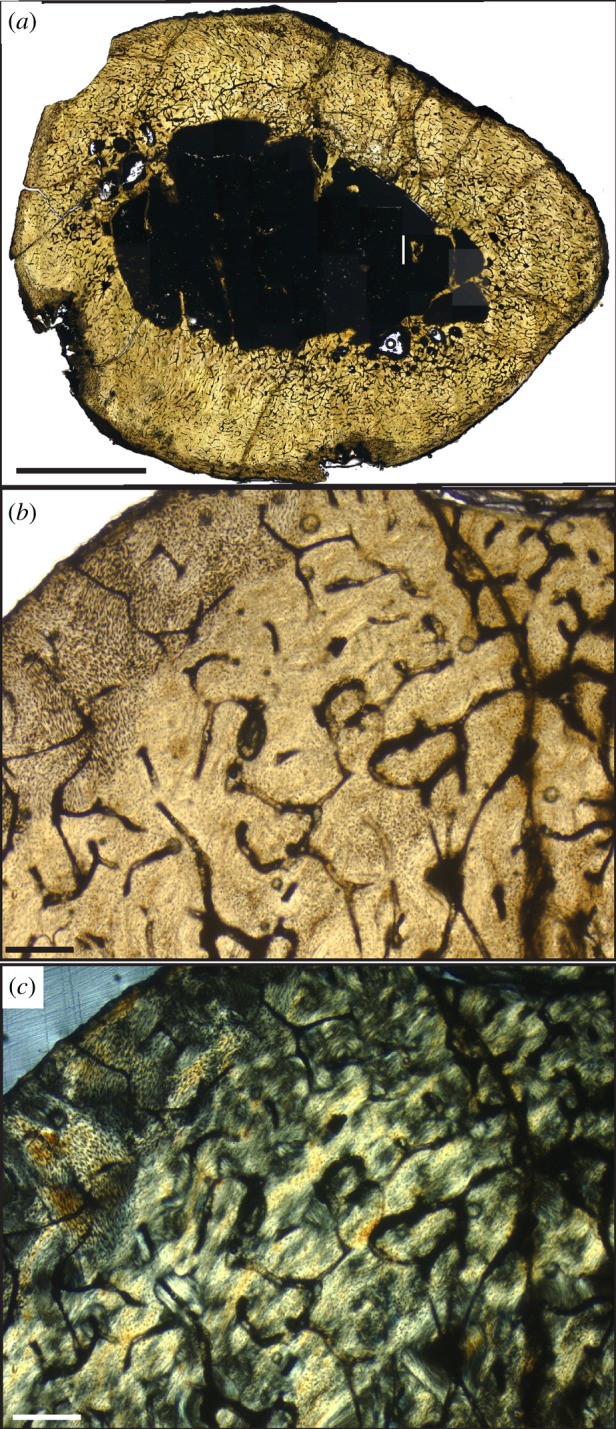
Bone microstructure of Devon Island cf. *Hesperornis* femur (NUVF 286). (*a*) Composite transverse section of femur showing shape and general histology patterns. Scale bar, 5 mm. (*b*) Close-up of cortical bone microstructure in plain light. Scale bar, 250 μm. (*c*) Same view as (*b*) but in cross-polarized light to show collagen fibre orientation. Scale bar, 250 μm.

All *Hesperornis* bones analysed in this study have cortical bone tissues comprised fast-growing, well-vascularized woven bone tissue with longitudinal and reticular primary vascular canal patterns (figures 2–4) (terminology following [27]). This bone is identified as fibrolamellar based on the presence of primary osteons with abundant osteocytes embedded in a woven-fibred bone tissue. This bone tissue is consistent with previously published descriptions of the bone histology of *Hesperornis regalis* specimens from the Niobrara Chalk of Kansas [50,58]. No LAGs or cyclic growth marks were observed in any *Hesperornis* specimens.

In general, the microstructure of *Hesperornis* bones is characterized by three layers (from endosteal to periosteal margins): a layer of endosteal lamellar bone (ELB) along the innermost cortex, a well-vascularized fibrolamellar layer in the mid-cortex and an outer circumferential layer (OCL, *sensu* [59]) lining the periosteal surface (figure 2). However, whether all three layers are present depends on the ontogenetic stage of an individual at the time of death. Both the ELB and OCL are composed of lamellar bone with well-organized collagen fibres (bright areas under cross-polarized light; figures 3*c*,*f* and 4*c*), low or no vascularity and flattened (versus globular) osteocytes observed in the transverse plane. The ELB is usually thicker than the OCL, and both show a uniform change in collagen fibre orientation under polarized light compared with cortical bone (figure 3). The three layers of bone tissue in *Hesperornis* matches bone microstructure patterns in extant birds [4,31,58,60–62], as well as in fossil birds inferred to be physiologically similar to extant birds [52,58,60,63].

### 4.1 Kansas tibiotarsi

Longitudinal and reticular vascular canals dominate the fibrolamellar framework of the primary cortical bone of the Kansas *Hesperornis*tibiotarsi (YPM 1489, YPM 1491), though localized circumferential and radial canals are also present in the inner and middle cortex (figure 3*a*,*b*). Both tibiotarsi also have circumferential vascular canals in the outer cortex. An OCL is apparent around the periosteal surface of both tibiotarsi, where woven bone changes to lamellar bone with well-organized, transversely oriented collagen fibres, sparse and small longitudinal vascular canals and flattened osteocytes (figures 2 and 3*c*). A layer of ELB is evident along the medullary cavity of both specimens, though the thickness of this zone varies within and between tibiotarsi. The ELB is largely avascular with well-organized collagen fibres and flattened osteocytes; these features are similar to those of the OCL. Secondary osteons are scattered through the cortex of the tibiotarsi, but extensive secondary reconstruction is not common. The medullary cavities of both tibiotarsi are completely open and devoid of spongiose bone (figure 3*a*).

### 4.2 Kansas femur

Like the tibiotarsi, the fibrolamellar bone of the mid-latitude *Hesperornis* femur is characterized by longitudinally oriented vascular canals, with some anastamosing canals comprising patches of reticular bone in the cortex (figure 3*d*,*e*). A few radial and circumferential canals are localized in the mid-cortex, but are not widespread. In some areas, abrasion along the periosteal surface has truncated primary and secondary osteons. However, where the periosteal surface of the femur is intact, a thin layer of lamellar bone with reduced vascularity is preserved, marking the presence of an OCL (figure 3*f*). An ELB deposit of inconsistent thickness lines the medullary cavity of YPM 1201. Taphonomic processes may account for some missing ELB, but this layer appears to be thinner in the femur than in the tibiotarsi. Secondary osteons are present, but the degree of secondary reconstruction varies throughout the cortex. The medullary cavity of the femur is also open and free of spongiose bone and bony trabeculae (figure 3*d*).

### 4.3 Devon Island femur

A vascular framework of longitudinal and reticular canals dominates the fibrolamellar cortical bone of the Devon Island cf. *Hesperornis* femur (figure 4*a*,*b*). Microbial invasion along the outer and inner margins of the femur shaft distorts the bone microstructure, but no distinct zone of lamellar bone is observed along the periosteal surface (figure 4*c*). Vascular canal density and size decrease towards the outermost cortical bone, though canals appear to be present at the periosteal surface in places. A thin ELB layer is observed along portions of the endosteal margin of the femur. Few secondary osteons are noted, but large erosion cavities with evidence of secondary bone deposition are present in the inner cortex. The medullary cavity of NUVF 286 is not filled with spongiose bone, but some unresorbed bony trabeculae extend into the cavity (figure 4*a*).

### 4.4 Ontogenetic stage

As a vertebrate with determinate growth reaches adult body size, bone deposition rate slows and the tissue is characterized by decreased vascularity and better organized (parallel-oriented) collagen fibres [28,29,47,57–59]. With the attainment of skeletal maturity, the OCL (or outer avascular layer, *sensu* [64]) develops and the presence of this layer can be used to determine whether an individual has reached adult body size [27,33,53,59,65,66]. Distinguishing skeletal maturity is especially useful for fossil specimens where the age of an individual at the time of death is unknown.

Histological analysis reveals the presence of an OCL in all three Kansas *Hesperornis* specimens (figure 3*c*,*f*), indicating that these individuals had reached skeletal maturity by the time of death. We therefore infer that these specimens represent adult birds. The Kansas specimens used in this study mark, to our knowledge, the first documentation of OCLs in *Hesperornis*; lamellar bone at the periosteal surface was not noted in *Hesperornis* bones analysed in previous studies [50,58]. Comparing OCL formation in extant birds, Ponton *et al.* [59] suggested that birds with larger body sizes have thinner OCLs relative to small-bodied birds. For example, as many ratites show little to no OCL formation [67,68], the authors [59] suggested that OCL formation may not occur in the largest extant birds (ratites). Subsequent studies have shown that an OCL is present along with LAGs in the large extinct moa [69], but these birds had prolonged growth and did not appear to reach skeletal maturity within a year, which indicates a different growth model than that of most extant birds. However, the presence of an OCL in YPM 1201, YPM 1489 and YPM 1491 shows that *Hesperornis*, a large bird without LAGs or evidence of prolonged juvenile growth, did deposit an OCL. This observation is consistent with the interpretations of authors of previous studies [50,58] that the absence of an OCL suggests that the hesperornithiform individuals examined had not attained skeletal maturity before death. It is also in agreement with the subsequent Cubo *et al.* [64] study showing that the presence and thickness of the OCL is not necessarily related to bone size.

In contrast to the Kansas specimens in this study, the Devon Island *Hesperornis* femur lacks a clear OCL (figure 4*c*). A thin layer of ELB is observed, though it is not as thick or well developed as it is in the Kansas specimens. The deposition of an ELB has been associated with later ontogenetic stages and reduced growth rates as skeletal maturity is approached [67]. Uniformity and thickness of this layer is also probably associated with the absorption of cancellous bone from the medullary cavity throughout ontogeny. Primary bone trabeculae extend into the medullary cavity in some areas of NUVF 286, signalling ongoing absorption along the endosteal surface. While the growth rate may be slowing, the medullary cavity is still changing shape, so the presence of an ELB is not necessarily associated with specific ontogenetic stages. Other features provide additional insights on ontogenetic stage. Secondary reconstruction is often found in juvenile and sub-adult bones [53], but secondary reconstruction plus reduced vascularity towards the outer cortex (evident in NUVF 286) is generally associated with slower bone deposition rates as an individual approaches adult body size [28,29,42,70]. Although the absence of an OCL in NUVF 286 suggests that skeletal maturity had not been reached, the advent of ELB development, decrease in vascularity towards the periosteal surface, nearly complete bone resorption in the medullary cavity, and secondary reconstruction through the cortex support a sub-adult ontogenetic stage approaching skeletal maturity at the time of death for the Arctic cf. *Hesperornis* specimen.

### 4.5 Pygoscelid bone microstructure

The penguin genus *Pygoscelis* includes three species—Adélie (*P. adeliae*), chinstrap (*P. antarctica*) and gentoo (*P. papua*)—with notably distinct biogeographic ranges, migratory behaviours and chick growth rates (table 2). Of the three species, gentoos have the widest (and most northern) distribution, the slowest growth constants (maximum slope of chick growth curve between 10 and 90% of adult size, *sensu* [43]) and a non-migratory lifestyle. By contrast, Adélies have the most southern (higher latitude) distribution, highest growth constants and are migratory. Chinstraps have an intermediate distribution, intermediate growth constants and are also migratory. Adélie and chinstrap penguins have been recorded over 3000 km [71–73] and 1000 km from their breeding colonies [74], respectively, during annual migrations. Gentoos are mostly sedentary, not typically venturing far from their breeding colonies during chick-rearing or winter [12]. The different migratory behaviours among pygoscelid penguins present an opportunity to test whether energy expenditures associated with long-distance migration is recorded in avian bone microstructure.

**Figure 5.**
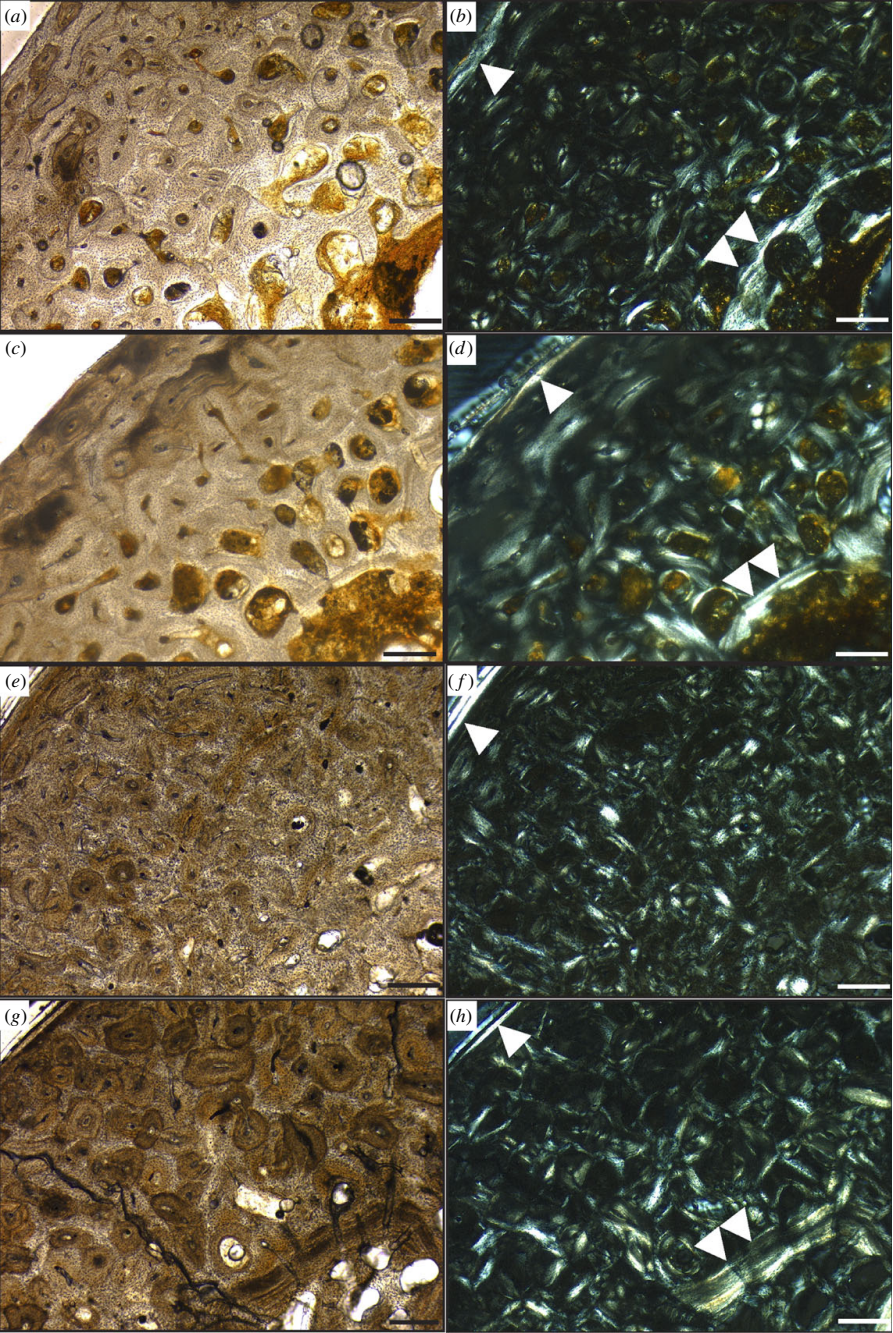
Bone microstructure of modern Adélie (UCM 104148) and chinstrap (UNCW 963) penguin long bones. All views show transverse sections through the bones. Adélie femur in (*a*) plain and (*b*) cross-polarized light. Adélie tibiotarsus in (*c*) plain and (*d*) cross-polarized light. Chinstrap femur in (*e*) plain and (*f*) cross-polarized light. Chinstrap tibiotarsus in (*f*) plain and (*g*) cross-polarized light. Single white arrows indicate the OCL and double white arrows indicate ELB. Scale bar, 250 μm for all images.

**Figure 6.**
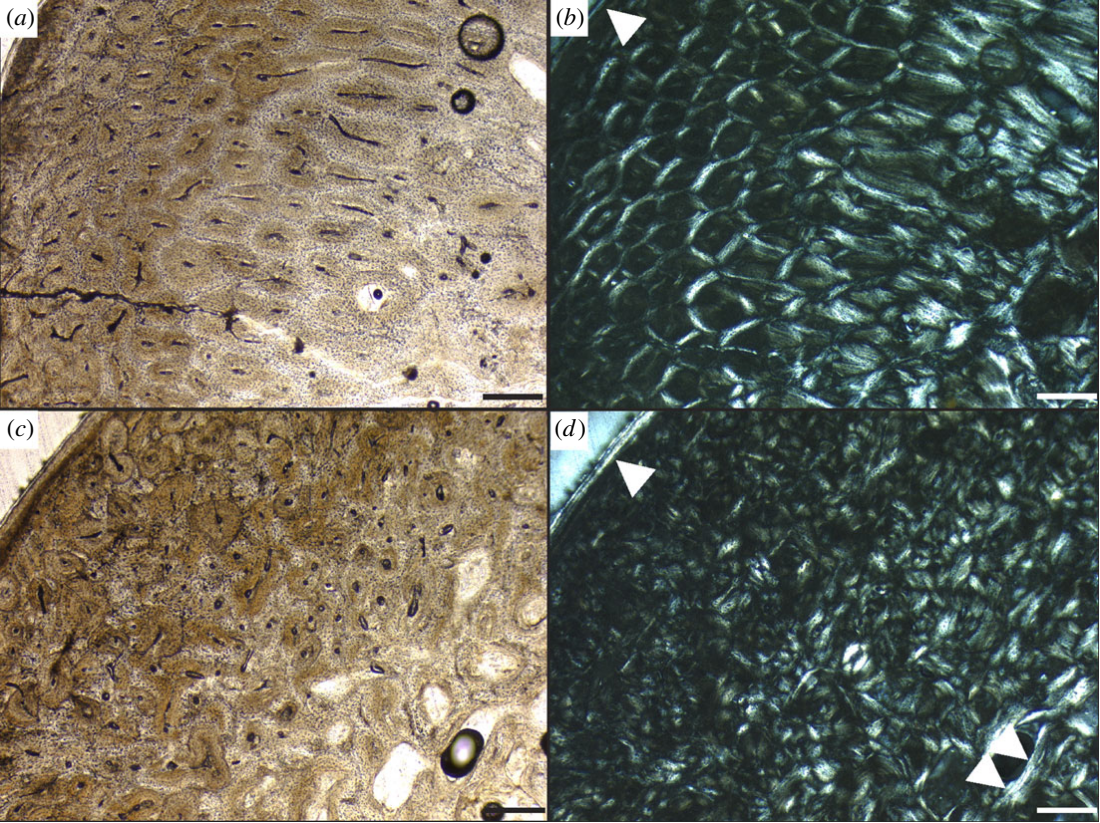
Bone microstructure of modern gentoo penguin (UNCW B980) long bones. Transverse section of gentoo femur in (*a*) plain and (*b*) cross-polarized light. Gentoo tibiotarsus in (*c*) plain and (*d*) cross-polarized light. Single white arrows indicate the OCL and double white arrows indicate ELB. Scale bar, 250 μm for all images.

Analysis of pygoscelid penguin bone microstructure shows that adult Adélie and chinstrap femora and tibiotarsi cortices are dominated by longitudinal canals with a few anastomosing canals (resulting in a reticular pattern) and moderate vascular canal density (figure 5 and table 2). Neither Adélie nor chinstrap leg bones contain any LAGs or annuli, nor is there evidence of cyclic growth in vascular canal patterns or collagen fibre orientation. Gentoo penguin femur microstructure differs from that of its congeners in being comprised more radial bone through the cortex, though they too lack LAGs (figure 6*a*,*b*; see also [4]). Additionally, adult gentoo leg bones have higher vascular canal densities than Adélies and chinstraps (table 2; [4]).

## 5. Discussion

Overall, few microstructural differences are evident between the Kansas *Hesperornis* and the Arctic specimen that cannot be attributed to ontogeny. Vascular canal patterns are similar in the mid- and high-latitude femora, with predominantly longitudinal and reticular vascular canals and localized areas of radial bone characterizing both specimens (figures 3 and 4). All bones also show moderate amounts of secondary reconstruction. The Devon Island femur has more reticular bone and slightly larger primary osteons compared with the Kansas femur and tibiotarsi, but this is probably related to individual variation and the sub-adult ontogenetic stage of the high-latitude specimen. The localized variation in vascular canal orientations among *Hesperornis* femora and tibiotarsi is unsurprising given studies on extinct and extant archosarus [27,28,33,53,61,66,75], noting variation in histological features. No evidence of growth marks, such as cyclic changes in vascular canal or collagen fibre orientation, LAGs or annuli are observed in any of the specimens (figures 2–4). Consequently, the bone microstructure of both high- and mid-latitude *Hesperornis* fossils indicates sustained, rapid growth through ontogeny with no episodic or cyclical growth patterns that would suggest accelerated and slowed/paused growth prior to skeletal maturity.

As noted above, bone histology has attracted recent attention as a potential proxy for determining whether populations undertook long migrations to avoid strongly seasonal environments. That is, it has been used to answer the question: were extinct taxa from high-latitude environments year-round residents or long-distance migrators? The former implies that they tolerated the extreme seasonality and periods of limited resources associated with polar light regimes and temperature fluctuations. The latter implies exposure to physiological stress caused by travel over long distances for long periods of time, although probably avoiding harsh environmental conditions.

Histological approaches to interpreting behavioural responses to polar conditions have largely focused on non-avian dinosaurs. Comparisons of dinosaur taxa from the same high-latitude deposit, as well as contemporaneous assemblages from different latitudes, have led to conflicting conclusions as to how bone microstructure relates to migration. These results largely rely on the presence of cyclic bone growth patterns; specifically, interpreting the occurrence of alternating zones of fast- and slow-growing bone, annuli and LAG deposits within the cortex. Cyclic growth marks are understood to be plesiomorphic or caused by seasonal and/or intrinsic cues [76,77], and there is currently no evidence based on extant organisms supporting environmental stress (which may be episodic rather than periodic) causing cyclic growth mark deposition in endotherms.

Several studies employing bone histology to decipher migratory behaviour conclude that regular, well-developed growth marks are more likely to be caused by overwintering in harsh environments rather than migration [28,37–39]. Erickson & Druckenmiller [39] compared histological patterns in ceratopsian bones from Alaska to related taxa from lower latitudes and concluded that cyclic growth marks are better developed in high-latitude specimens. This study, along with another on *Edmontosaurus* [37], suggest that microstructural differences between high- and lower latitude populations reflect different seasonal environmental stresses, ruling out that specimens from different latitudes represent members of the same populations that migrated along the WIS shoreline. It should be noted that the ontogenetic stages represented by some of the specimens in these studies are uncertain. Nevertheless, the findings are in agreement with Castanet *et al.*'s [28] overarching statement that strong seasonality produces well-developed growth marks in bone.

Tütken *et al.* [38] tackled the question of migration in fossil taxa by combining histology and stable isotope geochemistry in their analysis of contemporaneous dinosaurs from the Jurassic of China. They observed variable microstructure among bones of different taxa and made different behavioural interpretations for each species. One specimen with more histological and isotopic variation between zones in the bone tissue was interpreted as a year-round resident in a region with a strong monsoonal palaeoclimate. Conversely, another specimen with azonal bone uninterrupted by LAGs and less isotopic variability was interpreted as migratory. This implies that migration reduces the amount of seasonal environmental stress on an individual, resulting in an absence or reduction of cyclic growth marks. Again, however, the ontogenetic stages of some of the specimens in that study are unclear.

Contrary to the studies mentioned above, Hübner [40] concluded that the strong zonal bone development in large ornithopods in his study reflect the high physiological stress of migration. By comparison, he attributed the poorly developed, irregular growth marks noted in small ornithopods from the Late Jurassic/Early Cretaceous of Tanzania to physiological responses to episodic (versus periodic) environmental stresses such as droughts. He concluded that the irregular growth marks and small size of these ornithopods indicate that they were non-migratory.

Yet another view is presented in a recent histology study of Australian polar non-avian dinosaurs. Woodward *et al.* [41] found bone microstructure to be inconclusive for determining the effects of overwintering on bone growth. Chinsamy *et al.* [36] originally suggested that the presence of LAGs in polar theropods and the absence of LAGs in polar ornithopods indicated that the small Australian theropods hibernated to survive high latitudes' winters (both taxa are assumed to have been too small to migrate long distances). Subsequent research incorporating additional specimens revealed LAGs and cyclic bone growth in polar ornithopod bones that had not been previously documented [41]. The authors of this later study concluded that cyclic growth marks such as zones, annuli and/or LAGs in polar dinosaurs do not indicate hibernation or any patterns other than those typical of ontogenetic changes in dinosaurian bone microstructure. Instead, growth marks appear to be plesiomorphic features in archosauriform (including dinosaur) bone that reflect changes in growth rates through ontogeny [28,41,78]. Similar results from a recent study on ungulate mammals that showed that cyclical growth marks are deposited annually in high-, mid- and low-latitude ruminants [77] demonstrate that growth mark deposition is not conditional upon stressful environmental conditions.

Our study found no growth marks in the sampled *Hesperornis* leg bones regardless of ontogenetic stage or latitude (figures 2–4); this is in agreement with the results of previous studies [50,58]. Most extant birds are known to reach skeletal maturity within a year of hatching [29]. The known exception among extant birds is the kiwi, which deposits LAGs owing to an extended juvenile growth period [79]. The absence of LAGs and annuli in the bones of skeletally mature *Hesperornis* individuals implies that these birds reached skeletal maturity within 1 year of hatching. By attaining skeletal maturity within 1 year, most (if not all) primary cortical bone could be deposited by a bird's first winter or migration. This means appositional growth may have ceased by the time an annual growth mark would have formed or physiological stress from migration or overwintering could be recorded in bone microstructure.

That *Hesperornis* apparently attained skeletal maturity within 1 year suggests that growth dynamics were different in these birds than in non-avian dinosaurs. Nevertheless, their bone histology may reflect growth patterns that have implications for responses to winter conditions. Given their rapid growth, it is likely that these birds would have been physiologically able to endure long-distance migration by their first winter. This also indicates that they were large enough to survive the stresses of overwintering caused by low temperatures and reduced food resources. Rapid early ontogenetic growth rates and the attainment of adult body size within 1 year would have provided *Hesperornis* with an intrinsic means for success at high latitudes, whether they migrated or not. This interpretation is similar to Woodward *et al.*'s [41] conclusion that rapid growth with periods of growth cessation in Australian polar dinosaurs was an exaptation allowing for survival in extreme environments. It is also consistent with the fact that king penguin chicks in today's Antarctic region are known to undergo rapid growth and reach a size large enough to survive overwintering in the breeding colony [31,80]. The rapid growth phase of king penguins is reflected in their long bone histology [31].

### 5.1 Comparisons with extant *Pygoscelis* penguins

Essentially, no microstructural patterns are observed in the sampled bones of migratory Adélie and chinstrap penguins that can be correlated with the physiological stress of long-distance migration. As LAGs have been demonstrated to form annually in many tetrapods [28,76,77,81–83], the absence of growth marks together with the presence of an OCL indicates that these penguins attained skeletal maturity within 1 year of hatching (as discussed above for *Hesperornis*). The absence of growth marks also indicates that migratory behaviour is not recorded in the leg bones of at least these two extant migratory avian species in the form of LAGs or microstructural zones. Rapid skeletal maturity, the ability of individuals to avoid pronounced seasonal changes in temperature, photoperiod and food resources through migration, or different physiological adaptations related to rapid growth and smaller body size (table 2) may account for the absence. Adélies migrate their first year (as fledglings) [73], but it is presently unknown if they reach skeletal maturity by that time. This point requires further investigation, but if these birds actively deposit bone during their first migration, the absence of discernible changes in bone microstructure is significant for discussions regarding whether LAG formation can really be triggered by environmental conditions or physiological stress.

On the other hand, non-migratory gentoo penguin bones show a markedly different vasculature pattern than their congeners, most notably, the predominance of radial vascular canals. Radial vascular canal orientation reflects rapid bone growth in king penguins [31], and higher vascular canal density (table 2) also indicates higher bone growth rates in amniotes including birds [84,85]. This variation is much higher than between their other congeners, and also higher than the variability observed among *Hesperornis* specimens. The gentoo bones analysed were recovered from the southern portion of their range (tables 1 and 2), and Trivelpiece *et al.* [86] suggest that gentoos are physiologically adapted to the milder climates typifying the northern portion of their biogeographic range. Thus, it may be that the southern gentoos cope with overwintering in harsh environmental conditions by accelerating early ontogenetic bone development. If so, the high growth rates indicated by microstructural patterns in the analysed specimens may reveal mechanisms to cope with shorter seasons and harsher conditions [4,43,45]. As such, the overall high gentoo growth rates in southern populations may be related to the attainment of large body size during a short breeding season in a non-migratory species. Evidence for comparable rapid growth in the high-latitude *Hesperornis* bone was not observed.

## 6. Summary

This study considers the polar habitats of some hesperornithiform populations and offers insights on the life histories of these unique birds that thrived under climatic conditions distinctly different from those of today. Comparisons of modern pygoscelid penguin bones from closely related migratory (Adélie and chinstrap) and non-migratory (gentoo) species provide useful perspectives on the interpretation of bone microstructure in polar birds. Histological analyses of Adélie and chinstrap penguin bones reveal no cyclic patterns, uniform changes in microstructure or growth marks in the microstructure of these migratory species. This seems to support the current understanding that LAGs and other growth marks are triggered by environmental cues [76] and/or hormones [77] rather than environmental or physiological stress. However, high bone development rates are indicated by radial bone and higher vascular canal density in non-migratory gentoo penguins. The histological patterns of gentoos, compared to Adélies and chinstraps, probably reflect higher overall growth rates in gentoo populations in the southern portions of their biogeographic range where breeding seasons are shorter and winters are harsher [4,43,45]. Similar microstructure patterns are documented in king penguin chicks, which achieve exceptionally high growth rates to reach a body size large enough to survive their first Antarctic winter [31,80].

The histological patterns of high-latitude hesperornithiforms are indistinguishable from their lower latitude counterparts. All sectioned bones are dominated by microstructural patterns indicating continuous, rapid growth through ontogeny. High vascular canal density is dominated by longitudinal and reticular vascular canals. OCL s were documented for the first time, to our knowledge, in *Hesperornis* bones. However, no LAGs, other cyclic growth marks, or uniform changes in vasculature were observed. These observations indicate the attainment of skeletal maturing within 1 year of hatching.

Given comparisons to lower latitude specimens and modern avian analogues, the lack of growth marks, cyclic growth or significant radial bone deposits in the Devon Island *Hesperornis* are inconclusive in terms of interpreting migratory or overwintering behaviour in these extinct birds. Ultimately, results of histological analyses prompt two hypotheses regarding the life histories of high- and mid-latitude *Hesperornis* populations from the WIS. (i) *Hesperornis* migrated along the Seaway, but their migratory behaviour did not leave a record in their bone microstructure. Long-distance migration implies that these birds not only avoided Arctic winters, but that specimens collected from high- and mid-latitude localities were possibly members of the same populations. (ii) *Hesperornis* did not migrate, but overwintering in Late Cretaceous high-latitude environments did not leave a distinct record in their bone microstructure.

Different factors may account for the absence of histological patterns attributable to migration or overwintering in *Hesperornis*. Perhaps the most important consideration is that these birds reached adult body size within 1 year of hatching implies that most or all primary cortical bone could be deposited before undertaking long-distance migration or overwintering in a strongly seasonal climate. As a result, physiological stresses associated with these events may not be recorded in avian bone microstructure. More research is needed regarding whether migratory birds are skeletally mature before their first migration. Alternatively, *Hesperornis* may lack plasticity in their bone microstructure, meaning that changes in growth dynamics owing to migration or overwintering were not recorded.

It is also possible that Late Cretaceous Arctic climates were not severe enough to influence bone microstructure in *Hesperornis*. While there is evidence for seasonality [18,19,22] and below freezing temperatures [17,22,24,26] in the Late Cretaceous Arctic, the climate was much warmer and more equable than what characterizes today's high latitudes [14–18]. Not all overwintering high-latitude dinosaurs had distinctly different bone microstructure than their lower latitude relatives [41], and this may have also been the case for *Hesperornis*. Additional histological analyses of new hesperornithiform specimens may help tease out the causes and effects of behaviour and environment on their bone microstructure.

The continuing efforts to understand if and how bone microstructure patterns relate to migratory behaviour have resulted in numerous, often contradictory interpretations of migratory and non-migratory behaviour in fossil taxa [36–41]. Considering the hypothesized importance of growth marks and bone microstructure variations in interpreting behaviour in extinct taxa, the similar high- and low-latitude *Hesperornis* bone histology patterns provide new perspectives on using bone microstructure as a proxy for detecting evidence for migration or overwintering. It may be particularly important to consider whether fossil taxa reached skeletal maturity within 1 year like extant birds. Consequently, it seems that a multi-proxy approach may be the best way to realistically approach the question of migration in extinct taxa.

## Supplementary Material

Hesperornis and pygoscelid penguin femur and tibiotarsus measurements

## Data accessibility

Raw data is available in the electronic supplementary material at http://dx.doi.org/10.1098/rsos.140245. High resolution images and image metadata are available at Morphobank (www.morphobank.org) as Morphobank project P1270.
